# Video-assisted thoracic surgery (VATS) as a safe alternative for the resection of pulmonary metastases: a retrospective cohort study

**DOI:** 10.1186/1749-8090-4-13

**Published:** 2009-02-24

**Authors:** Marilee Carballo, Mary S Maish, Dawn E Jaroszewski, Carmack E Holmes

**Affiliations:** 1 Department of Surgery, David Geffen School of Medicine at UCLA, 10833 Le Conte Avenue, Los Angeles, CA 90095, USA; 2 Department of Surgery, Mayo Clinic Hospital, 5777 East Mayo Boulevard, Phoenix, AZ 85054, USA

## Abstract

**Background:**

VATS has become a preferred method for benign surgical conditions, yet still remains controversial for malignancies. The purpose of this study was to review our results of pulmonary metastasectomies using both conventional open thoracotomy and VATS techniques.

**Methods:**

This is a retrospective chart review of pulmonary metastasectomies performed from 1986 to 2006. The surgical approach used for the initial pulmonary metastasectomy was either open thoracotomy or VATS. Main outcomes were overall survival and recurrence free survival, evaluated using Kaplan Meier analysis. A non-inferiority margin was set at 0.2.

**Results:**

A total of 280 surgical procedures were performed on 186 patients. From 171 eligible individuals, 135 patients were treated with thoracotomy (82 M, 53 F; median age 49 years), and 36 with VATS (18 M, 18 F; median age 58.5 years). Primary cancers were mainly: 81 sarcoma (47%), 26 colorectal adenocarcinoma (15%) and 22 renal cell carcinoma (13%). Median postoperative follow was 26.2 months. The conversion rate was 10.3% and there were no cases of pleural cavity seeding. The 5-year overall survival rates were 58.8% for thoracotomy and 69.6% for VATS, with median overall survival of 53.2 months and 30.1 months, respectively (p = 0.03). The estimated difference in 5-year overall survival was 10.8%. Second occurrences were noted in 59 thoracotomy and 10 VATS patients. The 5-year recurrence free survival rates were 51% in thoracotomy and 67% in VATS (p = 0.27), with median recurrence free survival of 24.8 months and 25.6 months, respectively.

**Conclusion:**

In cases of pulmonary metastases, VATS is an acceptable alternative that is both safe and efficacious. Non-inferiority analysis of 5-year overall survival demonstrates that VATS is equivalent to thoracotomy. VATS patients also have a longer recurrence free survival. Based on our experience, it is permissible to use VATS resection in these circumstances: small tumor, fewer nodules, single lesion, age ≤ 53, unilateral, tumor size amenable to wedge resection, and non-recurrent disease.

## Background

Like other surgical specialties, thoracic surgery is moving towards less invasive techniques. In thoracic settings, a minimally invasive approach offers numerous benefits to the patient. Since its introduction in the early 1990s, video-assisted thoracoscopic surgery (VATS) has acquired widespread favor and is currently an essential part of thoracic surgeon armamentarium. VATS procedures are being used intensively to detect, diagnose and treat various benign conditions of the lungs, pleura, diaphragm, mediastinum, and upper GI tract.

Despite the controversy of using VATS to treat malignancies, anatomic pulmonary resection by VATS has become a widely accepted treatment for primary lung cancers as well as pulmonary metastases in the last decade [1]. VATS lobectomy with lymph node dissection has already gone well beyond the stage of an experimental technique and is on the way to becoming a standard procedure for stage I and II non-small cell lung cancer [2]. Although most pulmonary metastases are discreet peripheral nodules and can be completely removed by wedge resection, making them the perfect candidates for VATS, some issues exist concerning the safety of VATS – incomplete resection, port site and pleural cavity seeding [3]. But most often, VATS is criticized due to inability to perform thorough palpation of the entire lung, the well-established method to detect occult nodules missed on a conventional CT scan [4].

Although recent advancements in preoperative and intraoperative imaging allow detection of even non-palpable nodules [5], limited data directly comparing the oncological soundness of thoracotomy and VATS are available. In this study, we review our results of pulmonary metastasectomies using both VATS and conventional open thoracotomy techniques. We compare long-term clinical outcomes in order to determine whether or not VATS is of disadvantage to the patient from an oncologic standpoint. Given that the reported range of 5-year overall survival for patients with pulmonary metastases treated with VATS or thoracotomy varies from 30–50% among several independent studies [6-13], we also performed a non-inferiority analysis to compare the 5-year overall survival between the standard treatment (thoracotomy) and the newer treatment (VATS).

## Methods

### Eligibility Criteria

Patients with previous oncologic history were referred to our institution for surgical management of lung metastases. All patients who underwent a potentially curative resection of pulmonary metastases, had eradication of primary tumor, and absence or effective treatment of metastases at other organs – before or concurrent with pulmonary metastasis – were identified and included in this study. Patients were considered eligible for curative surgery on the basis of traditional staging (chest radiograph, bronchoscopy, thoracic/abdominal/brain CT). Surgeries performed for incomplete resection, biopsy-only and/or other diagnostic purposes were excluded.

### Study Design

A retrospective chart review of patients who underwent metastasectomies from January 1986 to November 2006 was conducted using the Patient Centric Information Management System at University of California, Los Angeles. This study reviews and compares the surgical treatment of pulmonary metastases by either traditional open thoracotomy or VATS. We also used a per-protocol analysis to analyze non-inferiority [14].

Patients were divided into 2 groups, based on the surgical approach used for the initial metastasectomy. Similar to the methodology of the International Registry of Lung Metastases, information was collected in four parts: (1) demographics, (2) oncological features of the primary neoplasm, (3) operative information of each metastasectomy, and (4) follow-up, including relapses of primary cancer, deaths and recurrences [15]. Maximum resection in one operation was counted as a type of resection, even if further wedge or segmental resections were performed. Pulmonary lesions were detected during routine follow-up by conventional CT scanner. The metastatic nature of lesions was presumed, but definitive diagnosis was made only after pathologic examination of resected specimens. Locoregional control was assumed.

### Baseline and Treatment Assessments

The primary outcome was overall survival (OS). The start point was taken as the date of resection of first pulmonary metastases (in cases of staged operation, the date of last operation). The Social Security Death Index (SSDI) was accessed for the patient status as of October 15, 2007. The endpoint was taken as either the date of death, or either October 15, 2007 or date of last follow-up, whichever was later, in cases where SSDI showed that a patient was alive. The secondary outcome was recurrence free survival (RFS), defined as date of initial metastasectomy to date of recurrence, instead of death. Disease free intervals (DFI) were defined as follows: (1) DFI-1: time between primary neoplasm and first pulmonary occurrence, (2) DFI-2: time between first and second occurrences, and so forth (i.e. DFI-3 to DFI-n).

To examine non-inferiority, the null hypothesis (H_0_) of the present study was that the absolute difference in 5-year survival between standard thoracotomy and VATS exceeds the non-inferiority margin (i.e. delta) of 0.20 [16-18]. The alternative hypothesis (H_a_) was that the absolute difference is smaller than delta. Power calculation estimated a total sample size of 186, based on a type I error (α) set at 0.05, type II error (β) set at 0.2, and delta 0.20 for the difference in 5-year survival. A total of 186 patients were recruited for this study.

### Statistical Methods

All data are reported as median or frequencies, using Mann-Whitney U test for continuous data, and Pearson χ^2 ^test or Fisher χ^2 ^exact for categorical data. Survival (OS and RFS) was estimated using Kaplan-Meier analysis. Actuarial 1-, 3-, 5- and 10-year survival rates were calculated, and corresponding survival curves were compared using the log-rank test [19]. Log-rank test was used in univariate analysis of prognostic influences of variables on survival. Cox Proportional Hazards model was used in multivariate analysis. Covariables were stepwise excluded if p > 0.2, and included if p < 0.15. A p-value < 0.05 was considered statistically significant [20]. All statistical manipulations were performed using MedCalc.

## Results

### Patient Characteristics

A total of 280 surgical procedures were performed on 186 consecutive patients. The majority of thoracotomies were performed from 1994–2005. We started to perform VATS metastasectomies in 1998, and as our experience with this approach was growing, the number of metastasectomies performed via this approach was increasing. Eleven VATS procedures were performed without curative intent – biopsy, hemothorax evacuation, talc pleurodesis, or pleural debris evacuation. Adequate information was available for the vast majority of patients. Only 15 (8%) were excluded from the present analysis, resulting in a total of 171 individuals evaluable for the study.

At the time of initial metastasectomy, tests for homogeneity comparing both groups showed no statistically significant p-values in most baseline patient and tumor characteristics (Table 1), except in primary tumor type, laterality of metastases, and nodal involvement. There were noticeably more of younger patients in the OPEN group. Sarcoma was more common in the OPEN group, while colorectal adenocarcinoma was more common in VATS. More tumors were unilateral and single nodular in the VATS compared to the OPEN group. PET use began in 1998 and was used in the evaluation of 32 patients (21 OPEN, 11 VATS). Most PET evaluations were done from 2002–2005 (n = 20).

**Table 1:** Baseline patient and tumor characteristics of pulmonary metastasectomies performed from January 1986 to November 2006

	**All (n = 171)**	**OPEN (n = 135)**	**VATS (n = 36)**	**p-value**†
Age (in years)*				< 0.01†
Median (Interquartile range)	53 (43–63)	49 (40–61)	58.5 (50.5–71)	
95% C.I.	49 to 56	47 to 55.25	52.85 to 66.29	

**Sex**				0.26
Female	71 (41.5)	53 (39.3)	18 (50)	
Male	100 (58.5)	82 (60.7)	18 (50)	

**Race**				0.06
Caucasian	140 (81.9)	112 (83.0)	28 (78)	
non-Caucasian	31 (18.1)	23 (17)	8 (22)	

**Comorbidities**				
Single	61 (35.7)	45 (33.3)	16 (44)	0.24
3 comorbidities	10 (5.8)	5 (3.7)	5 (14)	0.04†
Smoker	62 (36.3)	47 (34.8)	15 (41.7)	0.44

**Primary Tumor Type**				< 0.01†
Bladder	3 (1.8)	2 (1.5)	1 (3)	
Breast AC	9 (5.3)	5 (3.7)	4 (11)	
Colorectal AC	26 (15.2)	14 (10.4)	12 (33)	
Head/Neck	4 (2.3)	3 (2.2)	1 (3)	
Melanoma	14 (8.2)	6 (4.4)	8 (22)	
Renal cell	22 (12.9)	21 (15.6)	1 (3)	
Sarcoma	81 (47.4)	73 (54.1)	8 (22)	
Testicular	5 (2.9)	5 (3.7)	-	
Other	7 (4.1)	6 (4.4)	1 (3)	

**Adjuvant therapy**				
Chemotherapy	129 (75.4)	106 (78.5)	23 (64)	0.08
Radiation	73 (42.7)	54 (40)	19 (53)	0.19

**Laterality**				< 0.01†
Unilateral	124 (72.5)	90 (66.7)	34 (94)	
Bilateral	47 (27.5)	45 (33.3)	2 (6)	

**Magnitude**				0.06
Wedge resection	118 (69)	88 (65.2)	30 (83)	
All other	53 (31)	47 (34.8)	6 (16.7)	

**Number of lesions**				< 0.01†
Single	48 (28.1)	28 (20.7)	20 (56)	
2–5	94 (55)	79 (58.5)	15 (42)	
> 5	29 (17)	28 (20.7)	1 (3)	

**Metastatic Occurrences**				0.15
1	102 (59.6)	76 (56.3)	26 (72)	
2	38 (22.2)	31 (23)	7 (19)	
3+	31 (18.1)	28 (20.7)	3 (8)	

**Deaths**	74 (43)	65 (48)	9 (25)	0.02†

*All characteristics are expressed as no. (%), unless specified otherwise. Some covariables were grouped to preserve a 10% number of patients in each category for analysis. Non-Caucasian includes: Asian (10 OPEN; 7 VATS), Black (4 OPEN; 1 VATS) and Latino (9 OPEN; 0 VATS). All other includes: segmentectomy (17 OPEN; 4 VATS), lobectomy (29 OPEN; 2 VATS) and pneumonectomy (1 OPEN; 0 VATS). When PTT was categorized into carcinoma (includes bladder, breast, colorectal, melanoma, head/neck, renal cell, testicular, and 3 other type) and sarcoma (includes sarcoma and 3 other type), a statistical difference remained (p < 0.001). † Pearson χ^2 ^or Fisher exact; two-tailed p-value < 0.05 considered statistically significant.

### Complications

Among the 228 open surgeries performed, two patients required intraoperative transfusion, one of which was for disseminated intravascular coagulation. There were no intraoperative complications in any of the 52 VATS surgeries. The most common postoperative complications following thoracotomy were arrhythmia, transfusion and pleural effusion, whereas hydrothorax was common following VATS (Table 2). Atrial fibrillation was the most frequent arrhythmia (6 OPEN and 1 VATS). Among those who had thoracotomy, there was a single occurrence (< 0.5%) of each of the following postoperatively: hematoma, pleurocutaneous fistula, respiratory distress, post-thoracotomy syndrome, hemothorax and chylothorax.

**Table 2:** Postoperative complications for the two procedure groups, following 280 total pulmonary metastasectomies

	**OPEN (n = 228)**	**VATS (n = 52)**
Arrhythmia*	10 (4.4)	1 (1.9)
Pleural effusion	6 (2.6)	1 (1.9)
Transfusion	6 (2.6)	-
Prolonged air leak	5 (2.2)	1 (1.9)
Pneumothorax	4 (1.8)	-
Temporary vocal cord paralysis	3 (1.3)	1 (1.9)
Wound infection	3 (1.3)	-
Hydrothorax	2 (0.9)	3 (5.8)
Empyema	2 (0.9)	-
Post-obstructive pneumonia	2 (0.9)	-

*All characteristics are expressed as no. (%)

Conversion rate from VATS to thoracotomy was 10.3% (6 cases). Oncological reasons for conversion were: inability to perform resection of a difficult nodule due to insufficient exposure (n = 5) and finding of nodule(s) not shown on preoperative CT scan. In one patient, 1 of 2 nodules not seen on preoperative CT was detected intraoperatively by digital exploration. Following conversion, no additional nodules were found. Incomplete resection was seen in one VATS patient who did not meet inclusion criteria, and none had pleural cavity seeding.

Perioperative mortality (defined as death within 30 days from thoracic operation) was noted in one case. Following removal of a mucus plug by bronchoscopy, this patient was intubated for continued respiratory distress and required pressure support for increasing hypotension. He had continued hypoxemia, increasing acidemia and ultimately cardiac arrested. There were two other in-hospital deaths. One patient was admitted for respiratory distress and pneumonia, and expired four days later from respiratory failure. Another patient was admitted for brainstem hemorrhage from a metastatic lesion in the brainstem and cerebellum, and expired the next day from cardiopulmonary arrest. These two patients had their last pulmonary resection about eight months and 14 months prior to death, respectively. At completion of this study, 74 patients were dead (four OPEN and one VATS were thoracic deaths) and 97 alive (p = 0.88). The odds of death in thoracotomy was 2.79 times (OR [65 × 27/9 × 70]) the odds of death in VATS.

### Tumor Response

As of January 1994, at least one thoracic recurrence was seen in 69 patients, which were not all treated with the same initial procedure. Second occurrences were noted in 10 VATS patients, of which 6 were treated with thoracotomy. In the OPEN group, 47 were treated with thoracotomy a second time, and two with VATS. Third occurrences were noted in 3 VATS and 28 OPEN patients, of which 1 VATS and 25 OPEN patients were treated with thoracotomy. Most of the patients evaluated with PET had only one pulmonary metastases (n = 21), whereas 6 (19%) had two and 5 (16%) had three metastases. There is a possibility that using PET influences the outcome of patients differently than those who were imaged with chest radiograph or CT, although this was not assessed further. Tests for homogeneity among patients with recurrence (n = 69) showed no statistically significant difference in most baseline patient and all baseline tumor characteristics (Table 1). The odds of recurrence in thoracotomy was 2.02 times (OR [59 × 26/10 × 76]) the odds of recurrence in VATS.

### Time-to-Event Measures

#### Overall Survival

**Figure 1:**
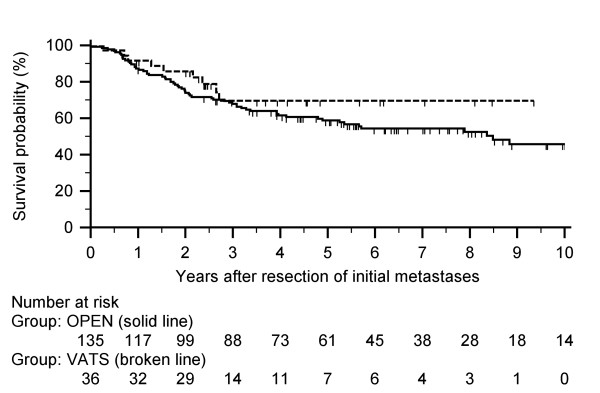
**Overall survival after initial metastasectomy for the two procedure groups**. Median overall survival was 47.3 months. The actuarial overall survival rates for VATS and open thoracotomy, respectively, are the following: 1-year at 91.7% and 87.4%; 3-year at 69.6% and 67.9%; and 5-year at 69.6% and 58.8%.

Median follow-up was shorter for VATS (30 months; 95% C.I. 27.13 to 47.66) than for thoracotomy (57 months; 95% C.I. 46.58 to 65.15) with the longest follow-up of 191.7 months. (p = 0.01). The median OS (n = 171) was 47 months (Table 3). The actuarial 5-year OS rate was 69.6% for VATS and 58.8% for thoracotomy (Figure 1 and Table 4); the estimated difference of 10.8% is less than delta. The lower one-sided 95% confidence limit was -0.036 (D - Z_α _× SED), which lies above the specified limit for Δ of -0.20 [16-18].

**Table 3:** Time-to-event measures for the two procedure groups

	All (n = 171)*	**OPEN (n = 135)**	**VATS (n = 36)**	**p-value†**
**DFI-1**	26.5 (14.1–67.5)	27.5 (14.5–74)	24.6 (14.0–55.1)	0.79
	23.54 to 33.02	23.99 to 34.4	14.26 to 40.05	

**OS**	47.3 (24–79.6)	53.2 (23.8–89.9)	30.1 (25.2–54.7)	0.03†
	38.46 to 57.97	44.73 to 64.13	27.13 to 47.66	

**DFI-2‡**	13.5 (7.1–31.1)	14.8 (7.2–34.3)	12.4 (5.6–13.5)	0.21
	10.33 to 18.86	9.32 to 22.44	2.45 to 18.06	

**RFS**	25.1 (10.3–57.9)	24.8 (9.8–59.9)	25.6 (12.4–45.9)	0.81
	20.11 to 31.76	19.72 to 36.2	13.41 to 32.22	

*First row: median (interquartile range); Second row: 95% C.I. around median.

†Two-tailed p-value < 0.05 considered statistically significant.

‡Second occurrence was seen in 59 OPEN and in 10 VATS patients.

**Table 4:** Univariate analysis of overall survival in all 171 patients (log-rank test)

	**n**	1-year*	**3-year**	**5-year**	**10-year**	**p-value†**
**Age (in years)**						0.03†
≤53	88	88.6 (76)	74.2 (57)	68.8 (42)	54.1 (10)	
> 53	83	88 (73)	62.8 (45)	51.7 (26)	42.8 (4)	

**Sex**						0.31
Female	71	85.9 (60)	73.8 (45)	64.6 (31)	51.8 (9)	
Male	100	90 (89)	65 (57)	57.7 (37)	44.4 (5)	

**Race**						0.20
Caucasian	140	88.6 (124)	66.4 (81)	59.1 (55)	44.4 (11)	
non-Caucasian	31	87.1 (25)	79.8 (21)	68.4 (13)	68.4 (3)	

**PTT**						0.02†
Carcinoma	87	93.1 (79)	74.8 (58)	69.2 (41)	57.3 (7)	
Sarcoma	84	83.3 (70)	62.5 (44)	51.4 (27)	39.2 (7)	

**DFI-1**						0.79
< 1 years	34	76.5 (26)	61.1 (16)	57.3 (14)	52.9 (2)	
1 to 5 years	92	90.2 (81)	70.3 (5)4	60.3 (34)	50 (7)	
> 5 years	45	93.3 (42)	71.1 (32)	63.5 (20)	44.5 (5)	

**Laterality**						0.40
Unilateral	124	91.1 (112)	72.4 (76)	63.3 (49)	46.4 (7)	
Bilateral	47	80.9 (37)	58.8 (26)	53.5 (19)	49 (7)	

**Magnitude**						0.17
Wedge	118	88.1 (102)	70 (71)	63.8 (56)	51.9 (12)	
All other	53	88.7 (47)	65.8 (31)	53.7 (12)	36.9 (2)	

**Number of lesions**						0.64
Single	48	91.7 (44)	71.8 (31)	66.9 (24)	47.8 (2)	
2–5	94	89.4 (82)	68 (53)	58.5 (32)	44 (6)	
> 5	29	79.3 (23)	65.5 (18)	56.4 (12)	56.4 (6)	

**Neoadjuvant**						0.10
No	99	85.9 (84)	62.9 (55)	54.4 (36)	46.4 (7)	
Yes	72	91.7 (65)	76.6 (47)	69.3 (32)	51.3 (7)	

**Procedure**						0.24
OPEN	135	87.4 (117)	67.9 (88)	58.8 (61)	45.8 (14)	
VATS	36	91.7 (32)	69.6 (14)	69.6 (7)	69.6 (0)	

*All survival rates are expressed as % (number at risk), unless specified otherwise.

† Two-tailed p-value < 0.05 considered statistically significant.

#### Recurrence Free Survival

**Figure 2:**
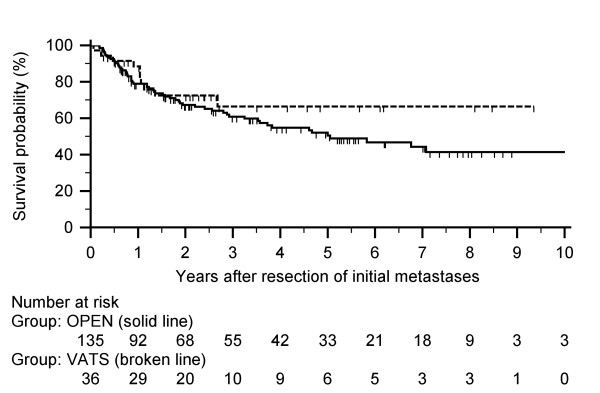
**Recurrence free survival after initial metastasectomy for the two procedure groups**. Median recurrence free survival was 25.1 months. The actuarial recurrence free survival rates for VATS and open thoracotomy, respectively, are the following: 1-year at 88.5% and 78.9%; 3-year at 66.5% and 60.9%; and 5-year at 66.5% and 50.5%.

Median DFI-2 was slightly longer for those in the OPEN group, and median RFS (n = 171) was 25 months (Table 3). Kaplan-Meier curve of RFS based on procedure is shown in Figure 2, and actuarial rates are shown in Table 5. The RFS was better for VATS at 5- and 10-years, although a significant difference was not seen. Most recurrences occurred within the first 2 years (37 OPEN, 8 VATS). Third thoracic occurrences were noted in 3 VATS (median DFI-3: 18 months) and 28 OPEN (median DFI-3: 11 months) patients. Fourth (n = 9, median DFI-4: 14 months), fifth (n = 3, median DFI-5: 19 months), sixth (n = 1, DFI-6: 14 months), seventh (n = 1, DFI-7: 21 months) and eighth (n = 1, DFI-8: 19 months) occurrences were noted in the thoracotomy group.

**Table 5:** Univariate analysis of recurrence free survival in all 171 patients (log-rank test)

	**n**	**1-year**	**3-year**	**5-year**	**10-year**	**p-value†**
**Age (in years)**						0.02†
≤ 53	88	73.8 (56)	55.9 (34)	44.9 (20)	37 (1)	
> 53	83	88.7 (65)	68.4 (31)	62.4 (19)	55.5 (2)	

**Sex**						0.09
Female	71	77.8 (47)	55 (25)	47.2 (15)	39.5 (2)	
Male	100	83.2 (74)	67.5 (40)	57.2 (24)	48 (1)	

**Race**						0.27
Caucasian	140	83 (102)	63.8 (54)	55.4 (33)	48.3 (3)	
non-Caucasian	31	72.1 (19)	54.7 (11)	42.2 (6)	21.1 (0)	

**PTT**						< 0.001†
Carcinoma	87	94 (76)	77.4 (45)	65.4 (28)	59.1 (2)	
Sarcoma	84	67.2 (45)	44.5 (20)	39.7 (11)	28.3 (1)	

**DFI-1**						0.79
< 1 years	34	84.9 (22)	58.2 (10)	58.2 (10)	58.2 (1)	
1 to 5 years	92	76.3 (62)	63 (37)	52.8 (20)	42.7 (1)	
> 5 years	45	88.4 (37)	62.8 (18)	49.4 (9)	35.1 (1)	

**Laterality**						0.03†
Unilateral	124	85.1 (96)	65.8 (51)	59.8 (31)	47.6 (1)	
Bilateral	47	69.3 (25)	51.2 (14)	34.9 (8)	34.9 (2)	

**Magnitude**						0.02†
Wedge	118	77.9 (80)	55.8 (42)	46.1 (28)	40 (2)	
All other	53	87.9 (41)	76.7 (23)	76.7 (11)	54.8 (1)	

**Number of lesions**						0.05
Single	48	91.4 (40)	69.3 (23)	62.7 (16)	51.2 (0)	
2–5	94	77.9 (65)	64.6 (34)	55.6 (19)	47.3 (3)	
> 5	29	72.9 (16)	40.1 (8)	27.5 (4)	27.5 (0)	

**Neoadjuvant**						0.63
No	99	78.4 (66)	62.2 (37)	51.6 (22)	41.3 (1)	
Yes	72	84.4 (55)	62.2 (28)	55 (17)	49.5 (2)	

**Procedure**						0.27
OPEN	135	78.9 (92)	60.9 (55)	50.5 (33)	41.5 (3)	
VATS	36	88.5 (29)	66.5 (10)	66.5 (6)	66.5 (0)	

*All survival rates are expressed as % (number at risk), unless specified otherwise.

† Two-tailed p-value < 0.05 considered statistically significant.

### Other Analyses

Univariate analysis of overall survival began with 9 potential predictors (Table 4). Age and PTT were the only predictors of OS. The 5-year OS was better in patients aged ≤ 53 and in those with carcinoma. The same potential predictors were used for univariate analysis of RFS (Table 5). Age, PTT, laterality and magnitude were the only predictors of RFS. Cox regression was then tested in all patients of the OPEN group (control) first; age and PTT were predictors of overall survival. When all patients of the VATS group were added and the procedures were included, age > 53 and sarcomatous PTT remained as negative predictors of OS (Table 6). Multivariate analysis of recurrence free survival in the thoracotomy group resulted in PTT and magnitude as predictors. When the VATS group was added and the procedures included, sarcoma and wedge remained as negative predictors of RFS (Table 6).

**Table 6:** Multivariate analysis of prognostic factors of overall survival and recurrence free survival (Cox Proportional Hazards)

	Exp (b)*	**95% C.I.**	**p-value**†
**Overall Survival, all patients**			
Age (1: above median; 0: below median)	2.21	1.33–3.67	0.002†
PTT (1: sarcoma; 0: carcinoma)	2.65	1.57–4.46	< 0.001†
Adjuvant therapy (1: yes; 0: no)	0.69	0.43–1.12	0.14

**Recurrence Free Survival, all patients**			
PTT (1: sarcoma; 0: carcinoma)	2.76	1.68–4.54	< 0.001†
Magnitude (1: other, 0: wedge)	0.54	0.29–0.98	0.05

*Exp (b) = Hazard Ratio

† Two-tailed p-value; < 0.05 considered statistically significant.

Overall survival analysis adjusted for age and RFS adjusted for magnitude were similar to their respective unadjusted analysis (data not shown). Overall and recurrence free survival changed when adjusting for primary tumor type. VATS patients with sarcoma had a better overall survival than all patients who had carcinoma. Patients with carcinoma had a better recurrence free survival; however, this was higher among thoracotomy patients.

## Discussion

### Key Results

The aim of the present study was to compare long-term clinical outcomes of the treatment of patients with pulmonary metastases to determine whether or not VATS is of disadvantage to the patient from an oncological standpoint. Our results show a 5-year overall survival rate of 70% in VATS patients. Open thoracotomy was taken as a reference treatment, with an expected survival of 50% since prior studies have demonstrated a 5-year survival ranging from 30–50% for the resection of pulmonary metastases. This study showed improved long-term survival in this group of patients treated with thoracotomy relative to previous studies [6-12], thereby supporting its consistent efficacy in the treatment of pulmonary metastases [17]. The Kaplan Meier analysis demonstrates that survival in this group of patients is comparable to the survival expected for lung cancer operations by thoracotomy [21].

Non-inferiority analysis suggests that the newer treatment (VATS) is not inferior to the standard treatment (thoracotomy). The 10% difference in overall survival was in favor of VATS, although this was not significantly different from those metastasectomies done using thoracotomy. However, since the estimated 5-year survival difference in our study of 0.108 was less than the non-inferiority margin set at 0.20, the findings support the conclusion that the 5-year overall survival rate using VATS is equivalent to thoracotomy for the treatment of pulmonary metastases. There were also no major differences in morbidity and mortality between both groups.

Selection of patients with the same prognosis is mandatory to accurately compare two therapeutic strategies. We took this into consideration to address the possibility that our analysis of non-inferiority is not valid given that the 36 patients in the VATS group had lesions associated with a more favorable prognosis. The two groups of this study were believed to be comparable in the majority of baseline patient and tumor characteristics with a few exceptions. Univariate and multivariate analyses demonstrated that age and primary tumor type were the only predictors of survival; however, from the adjusted analysis, we can infer that the imbalances in age and magnitude were not important and the groups are comparable in these features, and are unrelated to response to treatment. The only predictor which may influence the response to treatment is the primary tumor type.

### Limitations

We recognize that our sample size calculation (using α of 0.01 and Δ of 0.20) was more appropriately indicative in a per-protocol (PP) analysis, which requires fewer patients than an intention-to-treat (ITT) analysis. Despite the marginal difference between the computed size and the evaluable patients, PP analysis is preferable since ITT tends to bias towards making two treatments look similar [14]. For future investigations, the risk of Type I error can be minimized by setting α below the standard 0.05, or choosing a smaller non-inferiority margin [17].

This study is also a retrospective investigation. A prospective randomized controlled trial on a large scale is necessary to reach definitive conclusions regarding the efficacy of VATS for the treatment of pulmonary metastases relative to other techniques. However, the possibility of designing a three arm non-inferiority trial is limited by the controversy of using surgical placebos. We feel that non-inferiority of VATS over thoracotomy was effectively demonstrated without the use of a placebo. In this study, VATS was compared to an efficacious therapy [6,7,11], and VATS was believed to be comparable to thoracotomy. This permits basing efficacy conclusions of VATS in this study [22]. We also recognize the possibility of bio-creep and recommend that future trials exercise caution in the selection of an appropriate comparator therapy and non-inferiority margin.

The present study may be subject to pretreatment selection bias given that group assignment was determined through a shared decision made between physician and patient. This can produce misleading estimates of the size and direction of treatment effects [23]. For example, most of the patients selected for open thoracotomy were those with sarcoma and multiple lesions; these are associated with higher risk, suggesting that surgeon was selecting. Furthermore, the smaller number of patients available who had VATS may further exaggerate the better outcomes. Despite these possibilities, multivariate methods were used to reduce confounding and both groups were believed to be comparable. The increasing use of VATS will yield more cases to evaluate in future comparative studies over a considerable length of time.

### Interpretation

The present study demonstrates a favorable outcome in 5-year and possibly 10-year survival rates that will hopefully contribute to VATS gaining popularity in becoming a standard procedure. This is an interim evaluation until further follow-up can be obtained and reported. Although there is a difference in median follow-up, survivorship analysis allows for estimated survival rates to be more reliable, even when lengths of follow-up are not equal at any given date. The 10-year survival must be interpreted with caution since the number of subjects at risk at this time is small; however, the study adequately accounts for survivorship in both groups for at least 6 years after the start date.

The improved long-term survival seen in the present study relative to previous studies could be explained by a number of factors. The study includes patients from a single institution with extensive experience in treating pulmonary metastases, as well as a high volume of cases. Improvements in our ability to detect the progression of this disease may have occurred, as well as refinements in surgical techniques. The 5-year survival being better for VATS than thoracotomy could also be explained by the possibility of completer node clearance as a result of a clearer view and superior spread with VATS.

Altogether, among the prognostic variables tested in univariate analysis age, magnitude and primary tumor type were poor predictors of overall or recurrence free survival. After comparing the unadjusted and adjusted survival curves, primary tumor type remained as the only predictor that may influence the response to treatment. Furthermore, cancers known to be associated with poorer survival, such as testicular and melanoma, were all grouped into sarcoma. Most thoracotomy patients had sarcoma, which could explain the relatively lower overall and recurrence free survival compared to VATS. Patients with carcinoma had better overall and recurrence free survival, except for VATS patients with sarcoma who had the highest overall survival.

Although age does not seem to have a prognostic influence on overall survival in previously published reports [6,8,10,12,13,15], this study demonstrates that age > 53 had a negative influence on overall survival. This could be explained by the fact that these patients altogether are closer to death in the timeline of their cancer, and they will die sooner. On the other hand, Welter and associates found a survival advantage for elderly patients [24]. The difference in median age at the time of initial metastasectomy might be explained by the fact that younger patients can tolerate an open procedure better-better respiratory reserve and better health condition overall. Also, older patients are not offered open thoracotomy simply because they will not live long enough to benefit from the procedure. In the cases of younger patients, surgeon might offer open approach to ensure extra exploration (given the patient can tolerate it).

Overall survival might be expected to be better in the younger thoracotomy patients, especially since most VATS patients were older than the median age. The survival analysis adjusted for age showed that overall survival remained higher in VATS patients aged > 53, although this was not significantly different compared to the thoracotomy subgroup. We have not found an explanation for this in the literature. The favorable survival in this subgroup of VATS patients might also be explained by having more patients with unilateral and single nodular disease.

Longer DFI seems to be a favorable prognostic factor [7,15,24], although this study did not demonstrates DFI as a predictor of survival. The most favorable time interval cannot be clearly established since patients with a DFI-1 > 5 years had a better 1-, 3- and 5-year overall survival, yet a poorer 10-year overall survival rate. We found that the length of DFI-1 is associated with the length of DFI-2 in only one study [7]. This study does not demonstrate this association, and the use of VATS did not negatively impact DFI-2. Median DFI-2 was slightly shorter in VATS, although not statistically significant. There was no major difference in median recurrence free survival, although recurrences were seen less often with VATS patients, and the recurrence free survival at 5-years was much better than in the open thoracotomy group.

We did not take into account to location or tumor size, but our experience leads us to believe that metastases located in the hilar region should be treated with thoracotomy and larger tumors should be treated with resection that optimizes complete removal. We did consider magnitude of resection. Wedge resection was associated with a poor RFS in univariate analysis, which was also the case in multivariate analysis. This information may have skewed in favor of the other procedures because this included patients with segmentectomy, lobectomy and pneumonectomy. Furthermore, the single patient with pneumonectomy also had 100% OS and RFS. Lastly, since most patients with recurrence in both groups were treated with wedge resection, this may not have been the most ideal resection for the patient regardless of the type of surgery done.

One of the general goals of surgical oncology is complete removal of all metastatic deposits. In the International Registry of Lung Metastases, complete removal of all metastatic deposits was associated with long-term survival [15]. The results of this study are in agreement with published reports. Complete removal was associated with a 5-year overall survival of 70% in VATS and 59% in thoracotomy. In the thoracotomy group, there was one case where the pathology report showed some positivity at the margin, and one case with gross residual disease. Both occurred at the time of second occurrence, and neither had a third metastatic occurrence.

Some issues exist concerning the safety of VATS. Prior studies have reported conversion of VATS to thoracotomy due to incomplete fissures [25]. In this study, patients were not converted for this reason. Other issues include incomplete resection, and port site and pleural cavity seeding [3]. Incomplete resection was seen in only 1 VATS patient and this patient did not meet inclusion criteria. There were no cases of pleural cavity seeding in this study. There was only a single case where 1 of 2 nodules was not shown on preoperative CT, subsequently detected intraoperatively by digital exploration. Overall, the use of VATS did not appear to compromise the safety of the patients in this study.

## Conclusion

This study demonstrates equivalence in morbidity and mortality after resection of pulmonary metastases with either open thoracotomy or VATS. Furthermore, non-inferiority analysis of 5-year overall survival demonstrates that VATS is equivalent to thoracotomy. VATS patients also have a longer recurrence free survival. Based on our experience, we believe it is permissible to use VATS resection in these circumstances: small tumor, fewer nodules, single lesion, age ≤ 53, unilateral, tumor size amenable to wedge resection, and non-recurrent disease. Since this age group altogether showed a favorable recurrence free survival but poorer overall survival, whether VATS is also permissible in patients aged > 53 years is something to consider when selecting patients. Given the evidence of this study in conjunction with prior studies that have demonstrated the safety of VATS [21,26-28], we believe that VATS is an acceptable alternative for resection of pulmonary metastases that is both safe and efficacious under the recommended circumstances.

## Abbreviations

CT: computed tomography; DFI: disease free interval; DFI-1: DFI between primary tumor and first metastasectomy; DFI-2: DFI between first and second pulmonary occurrence; OR: odds ratio; OS: overall survival; PET: positron emission tomography; PTT: primary tumor type and RFS: recurrence free survival.

## Competing interests

The authors declare that they have no competing interests.

## Authors' contributions

MC participated in the design of the study, performed the statistical analysis and was involved in drafting the manuscript. MSM conceived of the study, participated in its design and coordination and was involved in drafting the manuscript. DEJ made a substantial contribution to the set up of the database and acquisition of data, as well as revised the manuscript critically for important content. ECH carried out analysis and interpretation of data, and revised the manuscript critically for important content. All authors read and approved the final manuscript.
